# Evaluating repellence properties of catnip essential oil against the mosquito species *Aedes aegypti* using a Y-tube olfactometer

**DOI:** 10.1038/s41598-024-52715-y

**Published:** 2024-01-27

**Authors:** Charles Batume, Ivan Mugeni Mulongo, Richard Ludlow, John Ssebaale, Peter Randerson, John A. Pickett, Ivan M. Mukisa, Simon Scofield

**Affiliations:** 1 Department of Entomology, Uganda Virus Research Institute (UVRI), Entebbe, Uganda; 2 Vector Genetics Laboratory, University of California Davis, Davis, CA USA; 3 School of Biosciences, Cardiff University, Cardiff, UK; 4 School of Chemistry, Cardiff University, Cardiff, UK; 5 CEMPOP Uganda, Ltd., Kampala, Uganda; 6 Department of Food Technology and Nutrition, Makerere University, Kampala, Uganda

**Keywords:** Biochemistry, Plant sciences, Secondary metabolism, Infectious diseases

## Abstract

The mosquito species *Aedes aegypti* (L.) is known to act as a vector in the transmission of various diseases, including dengue fever and yellow fever. The use of insect repellents is one of precautionary measures used to mitigate the risk of these diseases in humans by reducing mosquito biting. Nepetalactone, a potent natural insect repellent primarily found in catnip (*Nepeta cataria*) essential oil, has emerged as a promising candidate for mosquito repellence. Here, we evaluated the potential of catnip essential oil (> 95% nepetalactone) for use as a mosquito repellent. Using a Y-tube olfactometer and human hands as an attractant, we analysed the effectiveness of catnip oil at repelling the mosquito species *Aedes aegypti*. We tested a range of dilutions of catnip essential oil and found that concentrations as low as 2% were effective at repelling > 70% of mosquitoes for between one and four hours after repellent application. These findings suggest that nepetalactone could potentially be used as a natural, effective alternative to synthetic mosquito repellents, thereby offering protection against vector-borne diseases.

## Introduction

Insect-borne diseases pose a significant threat to human health, with mosquitoes (Diptera:Culicidae) acting as vectors of pathogens causing diseases such as malaria, dengue fever, filariasis, and West Nile virus^1^. Protecting against mosquito bites has traditionally involved limiting outdoor activities during peak mosquito activity, wearing protective clothing, and using insect repellents. While effective control measures, such as insecticide-treated nets and indoor residual sprays, have been implemented for indoor-biting mosquitoes in sub-Saharan countries such as Uganda, outdoor exposure to mosquitoes during work hours remains a challenge, particularly for *Aedes aegypti* (Linnaeus)*, Anopheles gambiae *sensu stricto (Giles)*, and Culex quinquefasciatus* (Say). Therefore, there is a need for an effective, user-friendly and affordable solution to prevent mosquito bites in these scenarios.

The most commonly used and effective mosquito repellent is the synthetic compound DEET (*N,N*-diethyl-3-methylbenzamide). However, concerns have been raised about the potential adverse effects of DEET, particularly in pregnant and lactating women, as well as in young children^2–4^. Despite these concerns, the centers for disease control and prevention (CDC) still recommend DEET for vector protection. While studies have shown that DEET can inhibit human acetylcholinesterase and modulate various receptors and ion channels, conflicting reports argue against significant adverse health effects^5,6^. Additionally, DEET is rapidly cleared from the body, unless ingested or used for prolonged periods^7–11^. Nonetheless, the cost of DEET remains a barrier for many individuals living in mosquito-prone regions like sub-Saharan Africa.

Exploring ethnobotanical resources has led to the identification of plant oils with insect-repellent properties, such as citronella, geraniol from lemongrass oil, and neem oil^12,13^. However, these natural alternatives have limitations, including shorter duration of repellence compared to DEET. Nevertheless, many studies have indicated that nepetalactone, an iridoid monoterpene found in catnip (*Nepeta cataria)* essential oil*,* can act as a highly effective mosquito repellent that is comparable to DEET in terms of its repellence properties^14–20^. Indeed, nepetalactone has been shown to have strong repellence properties against a broad range of other arthropods, including ticks^21^, bed-bugs^22,23^, dust mites^24^ and stable flies^25^.

Several studies have analysed the mosquito repellence properties of catnip oil-derived nepetalactone and its hydrogenated form, dihydronepetalactone^14–20^. These studies have used a range of methodologies, primarily utilizing in vitro attraction/ repellence assays using heat packs and blood samples, or in vivo assays utilizing shielded human subjects, and analysed the effects on several species of mosquito, including *Aedes aegypti* and *Anopheles gambiae s.s*. Most studies showed that catnip oil concentrations ranging from 10 to 50% provided effective repellence against mosquito landing, while others showed effective repellence with concentrations as low as < 1%^17^.

Catnip leaves have also been used in various applications, including tea, meat tenderizer, and folk remedies for fevers, colds, cramps, and migraines, suggesting that is safe for humans^26^. Safety assessments, such as acute oral, dermal, and inhalation toxicity tests, have deemed *N. cataria* essential oil safe for human use, as confirmed by the United States Environmental Protection Agency^27^. Additionally, Suschke et al. reported that catnip oil did not cause irritation when applied to human skin at a concentration of 25%^28^, further demonstrating its suitability as a topical repellent.

Building upon previous studies, this report demonstrates the suitability of the Y-tube olfactometer^29^ for conducting repellence assays to evaluate the repellence properties of essential oil from a *Nepeta cataria* chemotype (Chemotype A) which contains the 4a*S*, 7*S*, 7a*R* isomer of nepetalactone at a concentration of > 95%. We explored the effectiveness of catnip oil concentrations ranging from 0 to 100% in repelling the mosquito species *Aedes aegypti*, and found that concentrations as low as 2% provided effective repellence. By investigating the repellent properties of essential oils derived from catnip, this study aims to provide valuable insights into the development of effective and accessible mosquito repellents for populations in mosquito-prone regions. The affordability of locally sourced nepetalactone makes it a promising alternative or supplement to chemical-based repellents like DEET.

## Results

### Validation of Y-tube olfactometer function

**Figure 1:**
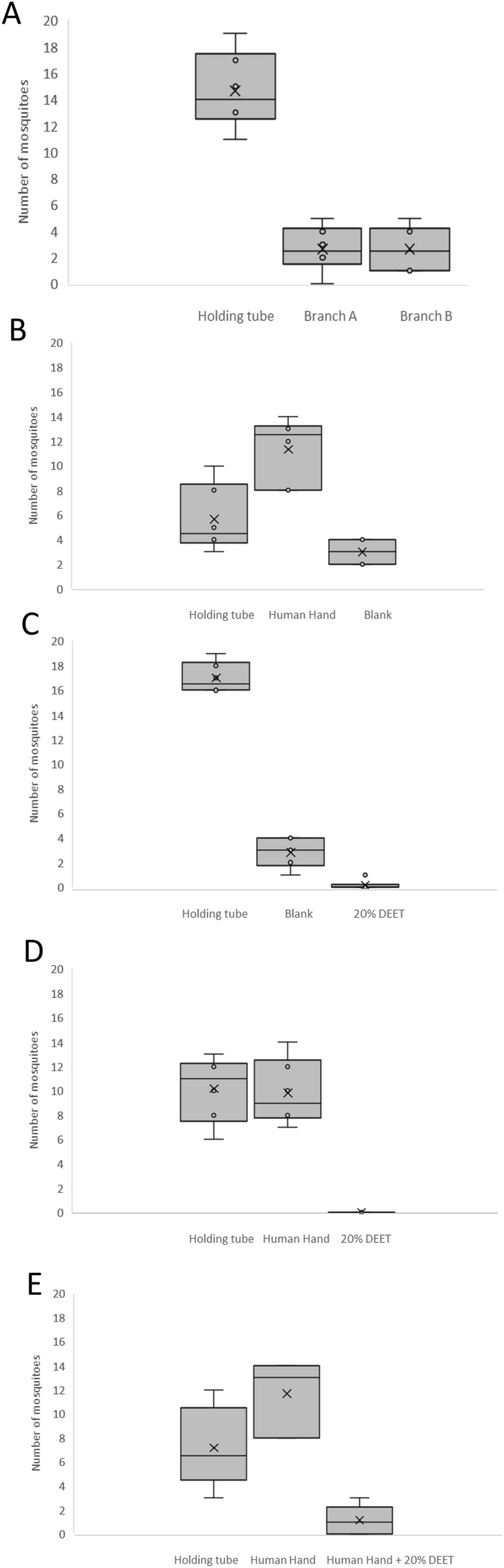
Validation of Y-tube olfactometer function. Box and whisker plots show mean number of mosquitoes per treatment and associated standard deviations. (**A**) Olfactometer bias experiment with no attractants or repellents. (**B**) Attraction assay using a human hand and empty olfactometer branch (blank). (**C**) Repellence assay using 20% DEET in one branch and an empty second branch (blank). (**D**) Attraction/repellence assay using human hand in one branch and 20% DEET in the other branch. (**E**) Attraction/repellence assay using human hand in one branch and human hand + 20% DEET in the other branch. All experiments were performed with six replicates of 20 mosquitoes per replicate. Branches for different treatments were alternated for experiments shown in (**B**–**E**). *t*-tests were performed as described in Materials and Methods and are shown in supplementary data.

Prior to testing the effectiveness of catnip oil as a mosquito repellent, we first evaluated our Y-tube olfactometer^29^ (Supplementary Fig. S1) for use in repellence assay experiments (Fig. 1; Supplementary Table S1). We first established whether the *Aedes aegypti* mosquitoes displayed a preference for a particular branch of the olfactometer, irrespective of the use of attractants or repellents. The distribution of mosquitoes in the olfactometer showed that an average of 13% travelled to the end of each collecting branch, while the remaining mosquitoes were distributed in the base leg or holding port (Fig. 1A; Supplementary Table S1). These findings indicate a random, uniform and repeatable distribution pattern of mosquitoes in both olfactometer branches. Thus, the Y-tube olfactometer is suitable for conducting unbiased repellence assays.

We next tested our apparatus to confirm that the presence of a human hand in one of the branches acted as an attractant to mosquitoes. In line with this, we found that an average of 57% of mosquitoes were attracted to the branch containing the human hand while 15% were found in the empty control branch and 28% remained in the holding port or base leg of the olfactometer (Fig. 1B; Supplementary Table S1).

We then performed a series of experiments to evaluate the performance of the mosquito repellent *N,N*-diethyl-3-methylbenzamide (DEET) using our olfactometer. We first placed an open vial containing 100 µl of 20% DEET in one branch and left the other branch empty. An average of 85% of mosquitoes remained in the holding tube/ base leg, while 14% were found in the empty branch and < 1% were found in the branch containing DEET (Fig. 1C; Supplementary Table S1). In the next experiment we placed a human hand in one branch as an attractant and a vial containing 100 µl of 20% DEET in the other branch. We found that 49% of mosquitoes were attracted to the human hand, while 51% remained in the holding tube/base leg. No mosquitoes were found in the branch containing DEET (Fig. 1D; Supplementary Table S1). Finally, we placed a human hand in one branch and a human hand with a vial containing 100 µl of 20% DEET in front of it in the other branch. An average of 62% of mosquitoes were found in the branch containing the human hand, while 33% remained in the holding tube/base leg and 5% were found in the branch containing the human hand + 20% DEET (Fig. 1E; Supplementary Table S1). These data confirm that human hands act as an attractant to *Aedes aegypti* mosquitoes and that DEET functions as an effective repellent using our Y-tube olfactometer and experimental methodology.

### Testing efficacy of commercially available repellents with the Y-tube olfactometer

**Figure 2:**
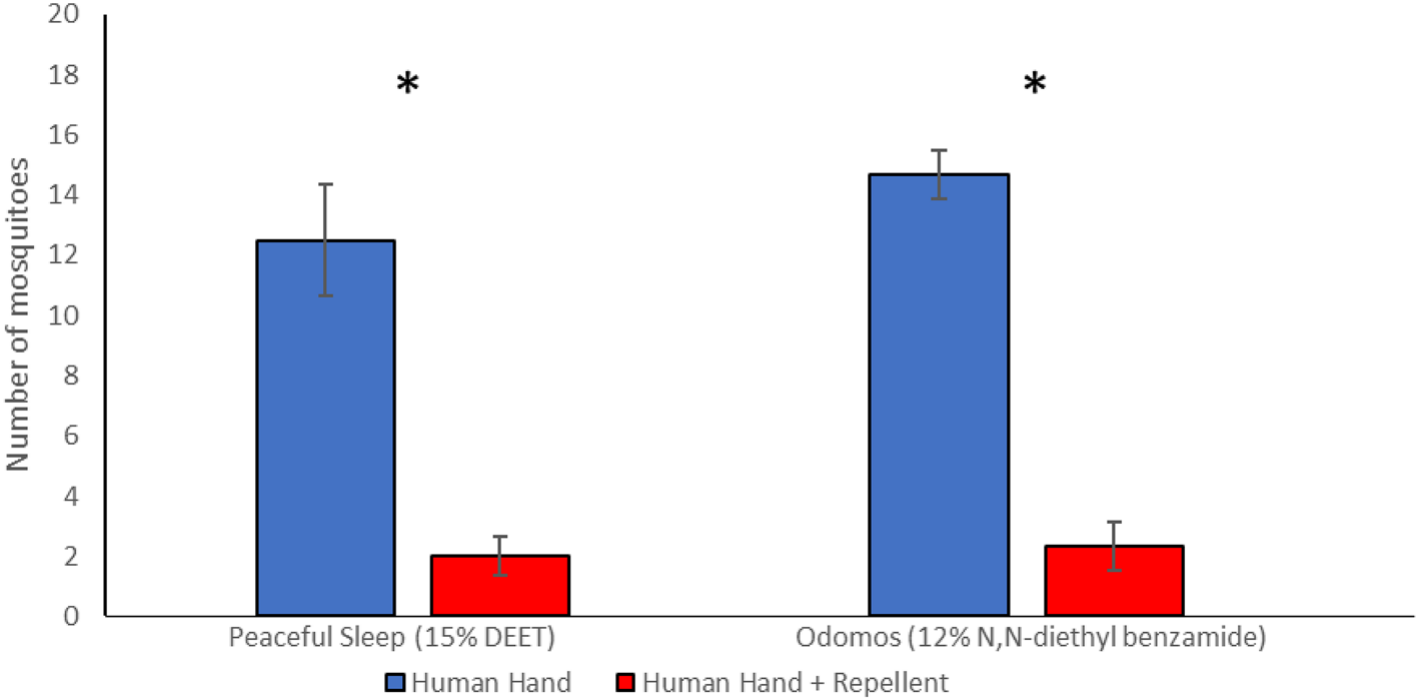
Repellence efficacy of commercially available mosquito repellents *Peaceful Sleep* (15% DEET) and *Odomos* (12% *N,N*-diethyl benzamide) using the Y-tube olfactometer. 18–20 mosquitoes were used for each replicate. A total of 6 replicates were used for each repellent. Error bars indicate standard deviations. *t* tests were performed as described in the Materials and Methods. Asterisks indicate statistical significance (*p* ≤ 0.01).

We tested our experimental setup and testing procedure with commercially available insect repellents *Peaceful Sleep* (15% DEET) and *Odomos*, which contains 12% *N,N*-diethylbenzamide, a compound closely related to DEET, that has been shown to be effective in repelling *Anopheles stephensi* and *Aedes aegypti* mosquitoes^30^. We found that *Peaceful Sleep*-treated human hands attracted an average of 14% of responding *Aedes aegypti* mosquitoes (i.e. only those mosquitoes that moved to either branch and did not remain in the base leg/holding port) while the untreated control hand attracted an average of 86% of mosquitoes (Fig. 2; Supplementary Table S2; Supplementary Fig. S2). Likewise, *Odomos*-treated human hands attracted an average of 14% of mosquitoes while the untreated control hand attracted an average of 86% mosquitoes (Fig. 2; Supplementary Table S2; Supplementary Fig. S2). These data confirm that our experimental design and apparatus can replicate the known mosquito repellence quality of DEET and *N,N*-diethylbenzamide, establishing that it is suitable for analysing the repellence qualities of other compounds.

### Catnip oil distillation and analysis

**Figure 3:**
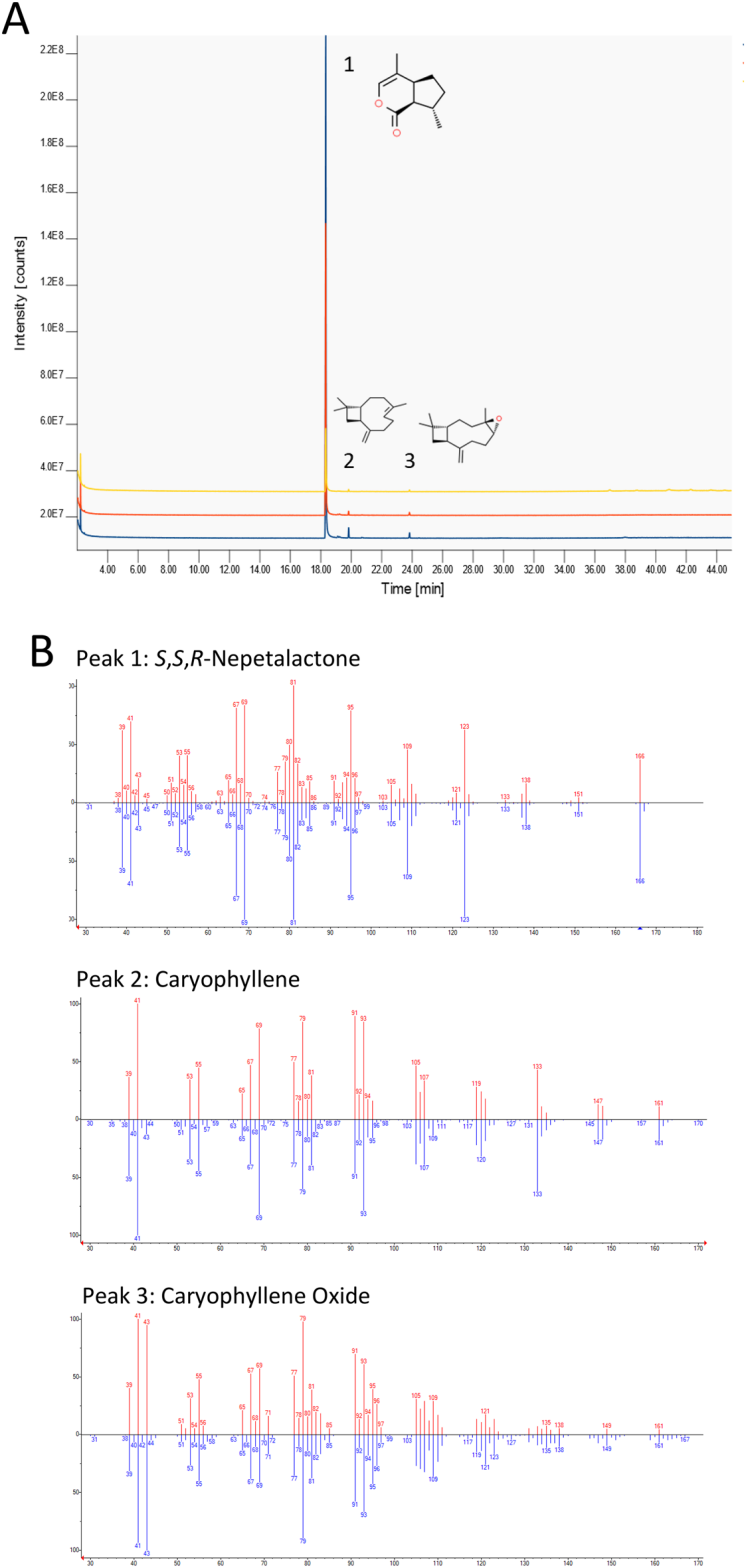
GC–MS analysis of *Nepeta cataria* Chemotype A essential oil. (**A**) Chromatograms obtained by GC–MS analysis of three independent replicates of *N. cataria* essential oil (superimposed, with base-lines adjusted by 1 intensity unit for ease of visualisation). Replicate 1 is shown in yellow, Replicate 2 in blue and Replicate 3 in red. Separate chromatograms are provided in Supplementary Data. (**B**) Head-to-tail plots of measured (red) vs library (blue) mass spectra using NIST mass spectral library search version 2.2. Y-axis = relative abundance of each ion, X-Axis = mass to charge ratio (m/z).

We obtained catnip essential oil by hydro-distillation of leaves, stems and flowers of a variety of *Nepeta cataria* designated as Chemotype A, which has previously been shown to contain the 4a*S*, 7*S*, 7a*R* isomer of nepetalactone^15^. Our GC–MS analysis showed that the oil contained ~ 97.5% nepetalactone,  ~ 1.5% caryophyllene and < 1% caryophyllene oxide (Fig. 3; Table 1). Hence, the oil contains a sufficient concentration of nepetalactone for use in mosquito repellence experiments.

**Table 1:** GC–MS analysis of *Nepeta cataria* chemotype A essential oil composition.

	(4aS, 7S, 7aR)-Nepetalactone %	Caryophyllene %	Caryophyllene oxide %
Replicate			
* Nepeta cataria* chemotype A replicate 1	96.76	1.78	1.46
* Nepeta cataria* chemotype A replicate 2	98.10	1.74	0.16
* Nepeta cataria* chemotype A replicate 3	98.14	1.02	0.83
Mean	97.67	1.51	0.82
Standard deviation	0.79	0.43	0.65

Oil distillation and GC–MS analysis were performed as per the “Materials and methods” section. Each replicate comprised a different batch of *N. cataria* tissue and was distilled separately. No other compounds passed the detection threshold.

### Testing efficacy of catnip oil in repelling *Aedes aegypti*

**Figure 4:**
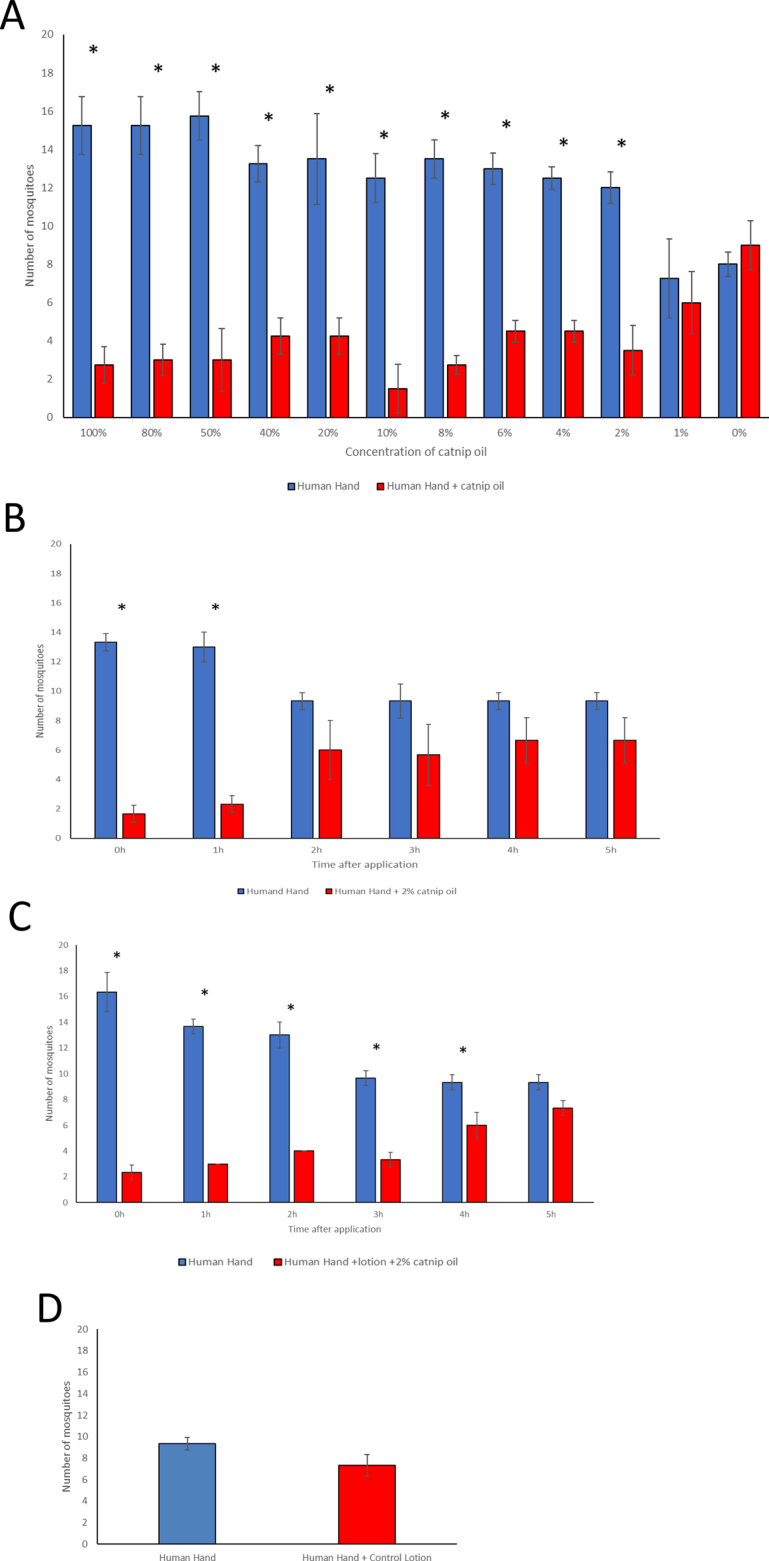
Evaluation of catnip oil as a mosquito repellent. (**A**) Number of mosquitoes attracted to a human hand (no catnip oil, blue bars) or human hand with catnip oil (red bars) applied at differing concentrations, diluted to the appropriate concentration in olive oil. 100% catnip oil had no olive oil diluent, 0% catnip oil was olive oil only. 20 mosquitoes were used for each replicate and 4 replicates were performed for each concentration. Asterisks indicate statistical significance using a *t*-test with a *p*-value of ≤ 0.01. (**B**) Residual activity of 2% catnip oil diluted in olive oil over a 5-h time-course post-application. 20 mosquitoes were used for each replicate and 3 replicates were performed for each concentration. Asterisks indicate statistical significance using a *t*-test with a *p*-value of ≤ 0.01. (**C**) Residual activity of hand lotion supplemented with 2% catnip oil over a 5-h time-course post-application. 20 mosquitoes were used for each replicate and 3 replicates were performed for each concentration. Error bars represent standard deviations. Asterisks indicate statistical significance using a *t*-test with a *p*-value of ≤ 0.05. (**D**) Number of mosquitoes attracted to the human hand vs human hand + control lotion lacking catnip oil. 20 mosquitoes were used for each replicate. A total of 3 replicates were performed. *t*-test was performed as per the “Materials and Methods”. No significant difference was observed.

We next investigated the effectiveness of different concentrations of catnip essential oil in repelling *Aedes aegypti* mosquitoes. We assessed the efficacy of a series of catnip oil dilutions ranging from 100% catnip oil with no diluent (olive oil) to 0% catnip oil with 100% diluent, using diluent alone as the control. We found that catnip oil at concentrations ranging from 100% to just 2% provided significant repellence of mosquitoes (*p* < 0.01), with the percentage of responding mosquitoes in the control branch ranging from 74 to 90%, and the percentage of mosquitoes in the branch containing repellent ranging from 26 to 10% (Fig. 4A; Supplementary Tables S3 and S4; Supplementary Fig. S3). Catnip oil diluted to 1%, or diluent alone, did not show any significant repellence properties. These data demonstrate the catnip oil is effective in repelling *Aedes aegypti* mosquitoes at a broad range of concentrations, with 2% representing the lower limit of efficacy.

### Assessing the duration of mosquito repellence using 2% catnip oil

Having established that 2% catnip oil provided substantial *Aedes aegypti* mosquito repellence, we next investigated the duration of repellence after the initial application. We tested a 2% dilution of catnip oil, again using olive oil as the diluent, and the effect of 2% catnip oil in a standard hand lotion formulation (see Materials and Methods for ingredients). We performed the repellence assay over a 5-h period and found that 2% catnip oil diluted in olive oil gave significant repellence (*p* < 0.01) for up to one hour after application, with 85% of responding mosquitoes found in the branch containing the human hand and 15% of mosquitoes found in the branch containing the human hand and 2% catnip oil one hour after application (Fig. 4B; Supplementary Table S5 and S6; Supplementary Fig. S3). Some repellence was observed at later timepoints, with ~ 60% of responding mosquitoes found in the branch containing the human hand and ~ 40% of mosquitoes found in the branch containing the human hand and 2% catnip oil, but these results were not statistically significant.

For 2% catnip oil added to a hand lotion, we observed significant repellence for up to 4 h after application to the skin (*p* < 0.01; though the 2-h timepoint was only significant using a *p*-value of *p* < 0.05: see Supplementary data) with 82% of responding mosquitoes found in the branch containing the human hand and 18% in the branch containing the human hand and 2% catnip lotion 1 h after application, and 61% and 39% respectively after 4 h (Fig. 4C; Supplementary Table S7 and S8; Supplementary Fig. S3).

No significant repellence was observed with lotion lacking catnip oil (Fig. 4D; Supplementary Table S9, Supplementary Fig. S3). Hence, 2% catnip oil has significant repellence properties for variable amounts of time after the initial application, depending on the diluent used and the method of application.

## Discussion

In this study we investigated the use of catnip essential oil derived from the herbaceous plant *Nepeta cataria* (a member of the Lamiaceae or mint family), as a repellent of the mosquito species *Aedes aegypti*, which acts as a vector for several infectious human diseases in sub-Saharan Africa and elsewhere. The active component in catnip oil, the iridoid monoterpene nepetalactone, has been shown previously to have potent repellence properties on several other hematophagous arthropod species^14–25^. Mechanistically, nepetalactone has been shown activate olfactory receptor neurons within the basiconic sensilla of the maxillary palps of *Aedes aegypti*^31^, and to act as an agonist for the chemical irritant receptor TRPA1 in the fruit fly *Drosophila melanogaster* and in *Aedes aegypti*^32^. Here, we used a Y-tube olfactometer and used established experimental protocols^29^ to assess mosquito repellence effectiveness of catnip oil comprising > 95% 4a*S*,7*S*,7a*R* nepetalactone, obtained by hydro-distillation of plant tissue from *Nepeta cataria* Chemotype A^15^ and analysed by GC–MS.

Our initial experiments validated our experimental design and apparatus, as we detected no inherent bias in the mosquito preference for a particular branch of the olfactometer, and we were able to demonstrate effective mosquito attraction using human hands and repellence using 20% DEET. We also showed that the application of two different commercial brands of insect repellent, one containing 15% DEET (*Peaceful Sleep*) and one containing 12% of the closely related compound *N,N*-diethylbenzamide (*Odomos*), acted to effectively repel mosquitoes using our experimental system, with ~ 86% of mosquitoes repelled. These data show that our repellence assay can recapitulate the known repellence properties of DEET and *N,N*-diethylbenzamide. Although DEET is normally considered a contact repellent, rather than a spatial repellent, our data show that mosquitoes were repelled from the olfactometer branch containing DEET, both when applied as a 20% solution and in the form of *Peaceful Sleep* (15% DEET). This is potentially attributable to the active airflow from the olfactometer branch ports into the holding port/ base leg within the relatively small environment within the olfactometer, suggesting spatial repellence at short distances. Although studies have suggested that DEET interferes with the perception of lactic acid and 1-octen-3-ol emitted by the host^33–35^, it has been shown that mosquitoes can directly detect sense DEET using olfactory receptor neurons in antennae, demonstrating that DEET can indeed be perceived directly in the vapour phase^36^.

Previous studies have shown the nepetalactone can actively reduce mosquito landing events using in vitro and in vivo methodologies such as blood samples, heat packs and human subject landing assays. For example, using hand-landing assays, Doraysamy et al. (2012) found that catnip oil used at concentrations of 10–50% gave repellence of 62–80% against *Aedes aegypti*, compared to 100% repellence when using DEET^16^. Similarly, Chauhan et al. (2005) found the nepetalactone was an effective repellent of *Aedes aegypti* using both in vitro assays and human attraction assays, though it was shown not to be as effective as DEET^18^. However, Reichart et al. (2019) found that 1% catnip oil extract, pure nepetalactone isomers or DEET were equally effective at repelling *Aedes aegypti*, and 10% solutions were able to sustain the repellence for 2-4h^17^.

The hydrogenated form of nepetalactone has also been shown to act as an effective mosquito repellent. Spero et al. found that a 15% dihydronepetalactone solution conferred repellence against *Aedes intrudens* Dyar for up to 4 h in the field^14^ and Feaster et al. found that effectively repelled mosquitoes at concentrations of 5–10% using both in vitro assays and human landing assays^20^.

Our study builds upon these findings by directly assaying mosquito repellence using the Y-tube olfactometer to provide a binary choice for landing and by using human hands as the attractant. We performed mosquito repellence assays using a range of different concentrations of catnip oil, diluted as appropriate in the olive oil diluent. We showed that concentrations as low as just 2% catnip oil provided effective repellence similar to that observed with higher concentrations of up to 100% catnip oil, and to the degree of repellence observed with 15% DEET. At concentration of 1% and 0% catnip oil, no repellence was observed, indicating that 2% is the lower limit for repellence, and that the olive oil diluent itself possesses no repellence properties.

We showed that 2% catnip oil provides statistically significant repellence for a period of up to 1 h when diluted in olive oil, and for up to 4 h when mixed with an unscented lotion that can be applied directly to human skin. This variation in repellence duration may depend on the differences between the olive oil and lotion diluents, or may reflect the different method of application, where application of lotion to the human hand may lead to greater volatility of nepetalactone. The use of just 2% catnip oil is far more cost-effective than using higher concentrations, and reduces the apparent strength of the odour as perceived by humans. This implies that, for people living in sub-Saharan Africa, 2% catnip oil can provide protection for the period spent undertaking chores in the evening, when many species of mosquito are most active. However, *Aedes aegypti* has also been found to bite during day, and therefore application of the repellent containing 2% catnip oil several times per day can be potentially as effective as the 15% catnip oil concentration tested by Spero et al.^14^.

## Conclusions

This study highlights the efficacy of catnip oil as a natural insect repellent, specifically for repelling *Aedes aegypti* mosquitoes, and the utility of the Y-tube olfactometer in conducting repellence trials. The results demonstrate that even at a low concentration of 2%, catnip oil provides significant protection for up to 4 h, making it a viable option for inclusion in consumer products like lotions, and may represent an effective, cheaper and more sustainable alternative to repellents such as DEET^37^. The catnip plant is easily cultivated in much of sub-Saharan Africa, especially in Uganda, and a number of indigenous catnip varieties exist which could be analysed and potentially used as a nepetalactone feedstock. Further research is warranted to validate these findings in different settings, such as in field trials employing human landing catch assays or arm-in-cage tests, and against other species of mosquitoes and other biting arthropods, enabling potential applications for mosquito-borne disease prevention to be explored.

## Materials and methods

### Rearing and feeding of *Aedes aegypti* mosquitoes

The mosquito repellence aspect of this project was carried out at Makerere University College of Food Science technology, Nutrition and Bioengineering, Kampala, Uganda. The colony of *Aedes aegyepti* was established by obtaining eggs and larvae from ovitraps set in the field (grassland) around Makerere University and rearing was conducted at the insectary at the Makerere University College of Food technology, Nutrition and Bioengineering. Traps containing eggs were brought back to the insectary facility and placed in distilled water to hatch. Larvae were reared in water at 27 °C under a 12-h day/night light cycle. After egg hatching, larvae were separated from the unhatched eggs and placed into fresh water. A small amount (about 10 mg) of tetramine fish food purchased from Tetra (Blacksburg, VA) was added to feed the larvae twice daily. Pupae were separated from larvae and placed into a (30 × 30 × 30) cm rearing cage where the pupae were allowed to mature into adults. These were given 5 days to mate. Mature females were visually identified, then separated out of the population by aspirating them into a separate rearing cage where they were given a 10% sucrose solution as an energy source. The method of rearing mosquitoes in the insectary followed well established protocols^38,39^. De-fibrinated bovine/ovine blood was used to feed the adult mosquitoes. Blood was stored at 4 °C and placed in warm water before feeding the mosquitoes. Mature females were maintained 27 ± 1 °C with 72–80% relative humidity in rearing cages until experimentation. Female mosquitoes that were nulliparous and 5–8 days post-emergence were used in the repellence experiments. Insectary hygiene, safety and security measures were implemented according to appropriate guidelines^38,39^.

### Purification and analysis of catnip essential oil

*Nepeta cataria* ‘Chemotype A’ seeds were sourced from CN Seeds (Cambridgeshire, UK). Essential oil from this *N. cataria* chemotype (Chemotype A) has previously been shown to contain > 91% 4a*S*, 7*S*, 7a*R* nepetalactone and has substantial insect repellent quality that has been demonstrated to work effectively on several species of mosquito, ticks and mites^15^. Plants were grown in 4L pots, in a potting mix consisting of 4 parts sieved compost to 1 part grit and 1 part John Innes No. 3. They were grown under heated glasshouse conditions and harvested at the point of floral opening. Plants were dried in a drying oven at 25 °C before being distilled. Catnip essential oil was obtained by hydro-distillation of dried mature aerial *Nepeta cataria* tissue using a Clevenger apparatus and a 5L round bottomed flask. 25–100 g of dried *N. cataria* tissue, 2.5L of water and 2 ml of hexane was boiled for 5 h and the oil distillate was collected and analysed by GC–MS. Three separate distillations were performed using variable *N. cataria* plant tissues (leaves, stems and flowers) grown independently. All plant research was conducted in compliance with international and UK guidelines. No endangered species were used in this research.

Essential oil samples were diluted by a factor of 100 in hexane (> 99%, Sigma-Aldrich). Samples were injected into a Thermo Trace 1300 gas chromatograph fitted with a Thermo TG-5MS column (30 m × 0.25 mm × 0.25 μm) and detected using a Thermo ISQ LT mass spectrometer. The injection port was operated at 200 °C, into which 1 μL of the sample was injected and loaded onto the column at a 1:5 split ratio, with the column being operated at 1 mL min^-1^ He carrier gas. The GC oven was run with a ramped temperature profile; initial temperature 50 °C for 2 min, ramp at 5 °C min^–1^ to 230 °C and held for 12 min. The mass spectrometer was operated with a transfer line temperature of 250 °C, an ion source temperature of 230 °C and a mass scan range of 35–350, with a 2 min solvent delay.

GC–MS data were processed using Chromeleon (Version 7.2 SR4; Thermo Scientific, USA) and the deconvolution and integration of signal peaks occurred in AMDIS (NIST, 2014), using a custom retention-indexed mass spectral library as previously described^40^. Compounds scoring > 80% in both forward and reverse fit which also had a retention index match of ± 15 were included in the custom library as putatively identified. The integrated signal of all identified peaks was summed for each sample, and results expressed as relative abundance (%) of each compound within a sample.

### Repellence testing procedure

The effectiveness of catnip oil and the branded repellents in mosquito repellence was tested using a Y-tube olfactometer based on that described by Geier and Boeckh^29^ in accordance with WHO guidelines^41^ (Supplementary Fig. S1). Preference for the subject’s treated/untreated hands, positioned at the ends of the Y-tube branches, was measured^29,42^. The positioning of test compounds (left or right branch) was varied to eliminate possible biases derived from the apparatus itself. Commercially available brands of mosquito repellents were used to validate our testing apparatus and protocol. These were *Peaceful Sleep* (15% DEET) and *Odomos* (12% w/v *N*,*N*-diethylbenzamide).

Mosquitoes were starved for 3–4 h prior to performing each experiment. The olfactometer was cleaned with 70% ethanol before and between experiments. Repellence assays were performed between 8:00 am and 6:00 pm with presence of natural light. Dilutions of catnip oil were prepared using a micropipette at a range of concentrations ranging from 0 to 100% diluted as appropriate using olive oil as the diluent and are hereafter termed ‘repellent’ while the control olive oil is termed ‘diluent only’. The lotion for assessing the duration of repellence contained 2% catnip oil, petrolatum, dimethicone, glycerol, sunflower oil, olive oil, shea butter, methyl paraben, propyl paraben, cetyl alcohol and stearyl alcohol. A 100 μl sample of repellent or control mixtures (olive oil diluent or lotion lacking catnip oil) was applied to a 9 mm vial cap and placed in front of the human subject’s hand to assay repellence, or directly to skin in the case of lotion repellence duration experiments. For repellence assays, 20 adult female *Aedes aegypti* mosquitoes were placed in the central holding port of the olfactometer and allowed to acclimatize for 5 min after the airflow fan was engaged at a rate of 0.4 m/s. 100 µl of repellent mixture was placed in a 9 mm vial cap in one olfactometer branch port and 100 µl of control mixture in front of the other, and human hands were placed at each port of the olfactometer. The placement of repellent and control treatments was fully randomized. The holding port door was opened for 30 s allowing the mosquitoes to fly along the base leg and into the two branches. The trapping port doors were closed and the number of mosquitoes in each trapping port were counted, along with those that remained in the holding port or base leg. Mosquitoes found in the holding port/ base-leg were not counted in data presented in the main figures as these could have been damaged or otherwise incapable of responding. Data including these non-responding mosquitoes are presented in the Supplementary Data file. Trapping ports and the holding port were removed and cleaned between replicates, and airflow was engaged for 5 min before the next replicate. The percentage attracted to the treatment port was calculated by dividing the number of mosquitoes trapped in the treatment port by the total number of responding mosquitoes in the test. The mean percentage attraction was calculated from the responses of a minimum of three replicates of each treatment. Data were analysed using Microsoft Excel. Statistical significance (p ≤ 0.01 or p < 0.05) was determined using a paired 2-tailed *t*-test assuming equal variance in the data. Normal distribution of data was confirmed using a Kolmogorov–Smirnov Test with the online tool: (https://www.statskingdom.com/kolmogorov-smirnov-test-calculator.html).

### Supplementary Information

Supplementary Information.

## Data Availability

All data are provided in the Supplementary Information. The raw data generated as part of this study are available from the corresponding author upon reasonable request.

## References

[1] World Health Organization. Vector borne diseases. https://www.who.int/news-room/fact-sheets/detail/vector-borne-diseases (2020).

[2] McGready R, Hamilton KA, Simpson JA, Cho T, Luxemburger C, Edwards R, Looareesuwan S, White NJ, Nosten F, Lindsay SW (2001). Safety of the insect repellent N, N-diethyl-meta-toluamide (DEET) in pregnancy. Am. J. Trop. Med. Hyg..

[3] Chen-Hussey V, Behrens R, Logan JG (2014). Assessment of methods used to determine the safety of the topical insect repellent N, N-diethyl-meta-toluamide (DEET). Parasite Vectors.

[4] Legeay S, Clere N, Apaire-Marchais V, Faure S, Lapied B (2018). Unusual modes of action of the repellent DEET in insects highlight some human side effects. Eur. J. Pharmacol..

[5] Corbel V, Stankiewicz M, Pennetier C, Fournier D, Stojan J, Girard E, Dimitrov M, Molgó J, Hougard JM, Lapied B (2009). Evidence for inhibition of cholinesterases in insect and mammalian nervous systems by the insect repellent deet. BMC Biol..

[6] Shelomi M (2020). Who’s afraid of DEET? Fearmongering in papers on botanical repellents. Malar. J..

[7] Selim S, Hartnagel RE, Osimitz TG, Gabriel KL, Schoenig GP (1995). Absorption, metabolism, and excretion of N, N-diethyl-m-toluamide following dermal application to human volunteers. Fundam. Appl. Toxicol..

[8] Wu A, Pearson ML, Shekoski DL (1979). High resolution gas chromatography/mass spectrometric characterization of urinary metabolites of N, N-diethyl-m-toluamide (DEET) in man. J. High Resolut. Chromatogr. Commun..

[9] Fediuk DJ, Wang T, Chen Y, Parkinson FE, Namaka MP, Simons KJ, Burczynski FJ, Gu X (2011). Tissue disposition of the insect repellent DEET and the sunscreen oxybenzone following intravenous and topical administration in rats. Biopharm. Drug Dispos..

[10] Qiu H, Jun HW, Dzimianski M, McCall J (1997). Reduced transdermal absorption of N, N-diethyl-m-toluamide from a new topical insect repellent formulation. Pharm. Dev. Technol..

[11] Qiu H, Jun HW, Tao J (1997). Pharmacokinetics of insect repellent N, N-diethyl-m-toluamide in beagle dogs following intravenous and topical routes of administration. J. Pharm. Sci..

[12] Maia MF, Moore SJ (2011). Plant-based insect repellents: A review of their efficacy, development and testing. Malar. J..

[13] Dias CN, Moraes DF (2014). Essential oils and their compounds as *Aedes*
*aegypti* L. (Diptera: Culicidae) larvicides: Review. Parasitol. Res..

[14] Spero NC, Gonzalez YI, Scialdone MA, Hallahan DL (2008). Repellency of hydrogenated catmint oil formulations to black flies and mosquitoes in the field. J. Med. Entomol..

[15] Birkett MA, Hassanali A, Hoglund S, Pettersson J, Pickett JA (2011). Repellent activity of catmint, *Nepeta*
*cataria*, and iridoid nepetalactone isomers against Afro-tropical mosquitoes, ixodid ticks and red poultry mites. Phytochemistry.

[16] Doraysamy D, Mulyaningsih B, Ernaningsih E (2012). Repellent activity of catnip extract (*Nepeta*
*cataria* L,) against *Aedes*
*aegypti* mosquito as dengue vector. Trop. Med. J..

[17] Reichert W, Ejercito J, Guda T, Dong X, Wu Q, Ray A, Simon JE (2019). Repellency assessment of *Nepeta*
*cataria* essential oils and isolated nepetalactones on *Aedes*
*aegypti*. Sci. Rep..

[18] Chauhan KR, Klun JA, Debboun M, Kramer M (2005). Feeding deterrent effects of catnip oil components compared with two synthetic amides against *Aedes*
*aegypti*. J. Med. Entomol..

[19] Bernier UR, Furman KD, Kline DL, Allan SA, Barnard DR (2005). Comparison of contact and spatial repellency of catnip oil and N, N-diethyl-3-methylbenzamide (deet) against mosquitoes. J. Med. Entomol..

[20] Feaster JE, Scialdone MA, Todd RG, Gonzalez YI, Foster JP, Hallahan DL (2009). Dihydronepetalactones deter feeding activity by mosquitoes, stable flies, and deer ticks. J. Med. Entomol..

[21] González J, Lockhart A, Wu Q, Simon JE, Toledo A (2022). Repellency of novel catnip (*Nepeta*
*cataria*) cultivar extracts against *Ixodes*
*scapularis* and *Haemaphysalis*
*longicornis* (Acari: Ixodida: Ixodidae). Ticks Tick Borne Dis..

[22] Shi X, Wang C, Simon JE, Reichert W, Wu Q (2021). Repellency of novel catnip oils against the bed bug (Hemiptera: Cimicidae). J. Med. Entomol..

[23] Patel HK, Gomes EN, Wu Q, Patel N, Kobayashi DY, Wang C, Simon JE (2023). Volatile metabolites from new cultivars of catnip and oregano as potential antibacterial and insect repellent agents. Front. Plant Sci..

[24] Khan MA, Jones I, Loza-Reyes E, Cameron MM, Pickett JA, Birkett MA (2012). Interference in foraging behaviour of European and American house dust mites *Dermatophagoides*
*pteronyssinus* and *Dermatophagoides*
*farinae* (Acari: Pyroglyphidae) by catmint, *Nepeta*
*cataria* (Lamiaceae). Exp. Appl. Acarol..

[25] Zhu JJ, Berkebile DR, Dunlap CA, Zhang A, Boxler D, Tangtrakulwanich K, Behle RW, Baxendale F, Brewer G (2012). Nepetalactones from essential oil of *Nepeta*
*cataria* represent a stable fly feeding and oviposition repellent. Med. Vet. Entomol..

[26] WebMD Editorial Contributors. Catnip Tea: Are There Health Benefits? https://www.webmd.com/diet/catnip-tea-health-benefits. Medically Reviewed by Kathleen M. Zelman, RD, LD, MPH (2023).

[27] United States Environment Protection Agency online. Science review and human health risk assessment in support of the registration of the insect repellent refined oil of Nepeta cataria (TGAI), and two lotion end-use products. https://www3.epa.gov/pesticides/chem_search/cleared_reviews/csr_PC-004801_4-Oct-07_a.pdf (2007).

[28] Suschke U, Sporer F, Schneele J, Geiss HK, Reichling J (2007). Antibacterial and cytotoxic activity of *Nepeta*
*Cataria* L., *N*. *Cataria*
*Var*. *Citriodora* (Beck.) Balb. and *Melissa*
*Officinalis* L. essential oils. Nat. Prod. Commun..

[29] Geier M, Boeckh J (1999). A new Y-tube olfactometer for mosquitoes to measure the attractiveness of host odours. Entomol. Exp. Appl..

[30] Mittal PK, Sreehari U, Razdan RK, Dash AP, Ansari MA (2011). Efficacy of advanced Odomos repellent cream (N, N-diethyl-benzamide) against mosquito vectors. Indian J. Med. Res..

[31] Sparks JT, Bohbot JD, Ristic M, Mišic D, Skoric M, Mattoo A, Dickens JC (2017). Chemosensory responses to the repellent nepeta essential oil and its major component nepetalactone by *Aedes*
*aegypti* (Diptera: Culicidae), a vector of Zika Virus. J. Med. Entomol..

[32] Melo N, Capek M, Arenas OM, Afify A, Yilmaz A, Potter CJ, Laminette PJ, Para A, Gallio M, Stensmyr MC (2021). The irritant receptor TRPA1 mediates the mosquito repellent effect of catnip. Curr. Biol..

[33] Davis EE (1985). Insect repellents: Concepts of their mode of action relative to potential sensory mechanisms in mosquitoes (Diptera: Culicidae). J. Med. Entomol..

[34] Dogan EB, Ayres JW, Rossignol PA (1999). Behavioural mode of action of deet: Inhibition of lactic acid attraction. Med. Vet. Entomol..

[35] Ditzen M, Pellegrino M, Vosshall LB (2008). Insect odorant receptors are molecular targets of the insect repellent DEET. Science.

[36] Syed Z, Leal WS (2008). Mosquitoes smell and avoid the insect repellent DEET. Proc. Natl. Acad. Sci. U.S.A..

[37] Patience GS, Karirekinyana G, Galli F, Patience NA, Kubwabo C, Collin G, Bizimana JC, Boffito DC (2018). Sustainable manufacture of insect repellents derived from *Nepeta*
*cataria*. Sci. Rep..

[38] Malaria Research and Reference Reagent Resource Center (MR4). Methods in Anopheles Research. 4th edition, **12**:1–90 (2014).

[39] Eukubay A, Wtensai A, Eticha G, Nigatu W, Wayessa A, Mekuriaw W, Wuletaw Y, Hmariam A, Tesfaye F, Asfaw T, Kinfe E (2017). Anopheles Mosquito Rearing and Insectary Handling Guideline.

[40] Ludlow RA, Pacenza M, Chiappetta A, Christofides SR, Evans G, Graz M, Marti G, Rogers HJ, Müller CT (2021). Storage time and temperature affects volatile organic compound profile, alliinase activity and postharvest quality in garlic. Postharvest Biol. Technol..

[41] World Health Organization Guidelines for efficacy testing of spatial repellents (2013).

[42] Logan JG, Birkett MA, Suzanne JC, Stephen P, Nicola JS, Lester JW, Mordue AJ, Pickett JA (2008). Identification of human-derived volatile chemicals that interfere with attraction of *Aedes aegypti* Mosquitoes. J. Chem. Ecol..

