# Supplementary material for: Evaluating repellence properties of catnip essential oil against the mosquito species Aedes aegypti using a Y-tube olfactometer
Source: Sci Rep. 2024 Jan 27;14:2269. doi: 10.1038/s41598-024-52715-y (PMC10821859; doi:10.1038/s41598-024-52715-y)
Supplement: Supplementary file 1 — Supplementary Information. [file 41598_2024_52715_MOESM1_ESM.docx]

**Supplementary Table S1**. Validation of Y-tube olfactometer function. 20 mosquitoes were used for each replicate. 6 replicates were performed for each experiment. *t*-tests were performed as per the Materials and Methods.

**Supplementary Table S2**. Repellence efficacy of commercially available mosquito repellents *Peaceful Sleep* (15% DEET) and *Odomos* (12% *N,N*-diethyl benzamide) using the Y-tube olfactometer. 18-20 mosquitoes were used for each replicate. A total of 6 replicates were used for each repellent. *t*-tests were performed as per the materials and methods.

**Supplementary Table S3.** Evaluation of catnip oil as a mosquito repellent (only responding mosquitoes are shown). 20 mosquitoes were used for each replicate. A total of 4 replicates were performed for each concentration of catnip oil, except for 0% where 6 replicates were performed. *t*-tests were performed for human hand + diluent vs human hand + repellent as per the materials and methods.

**Supplementary Table S4.** Evaluation of catnip oil as a mosquito repellent, including non-responding mosquitoes. 20 mosquitoes were used for each replicate. A total of 4 replicates were performed for each concentration of catnip oil, except for 0% where 6 replicates were performed.

**Supplementary Table S5.** Evaluation of duration of 2% catnip oil as a mosquito repellent (only responding mosquitoes are shown). 20 mosquitoes were used for each replicate. A total of 3 replicates were performed for each timepoint after application of 2% catnip oil. *t*-tests were performed for human hand + diluent vs human hand + repellent as per the materials and methods.

**Supplementary Table S6.** Evaluation of duration of 2% catnip oil as a mosquito repellent, including non-responding mosquitoes. 20 mosquitoes were used for each replicate. A total of 3 replicates were performed for each timepoint after application of 2% catnip oil.

**Supplementary Table S7.** Evaluation of duration of lotion containing 2% catnip oil as a mosquito repellent (only responding mosquitoes are shown). 20 mosquitoes were used for each replicate. A total of 3 replicates were performed for each timepoint after application lotion containing 2% catnip oil. *t*-tests were performed as per the materials and methods.

**Supplementary Table S8.** Evaluation of duration of lotion containing 2% catnip oil as a mosquito repellent, including non-responding mosquitoes. 20 mosquitoes were used for each replicate. A total of 3 replicates were performed for each timepoint after application lotion containing 2% catnip oil.

**Supplementary Table S9.** Evaluation of repellence properties of control lotion formulation lacking catnip oil, including non-responding mosquitoes. 20 mosquitoes were used for each replicate. A total of 3 replicates were performed. T-test was performed as per the Materials and Methods.

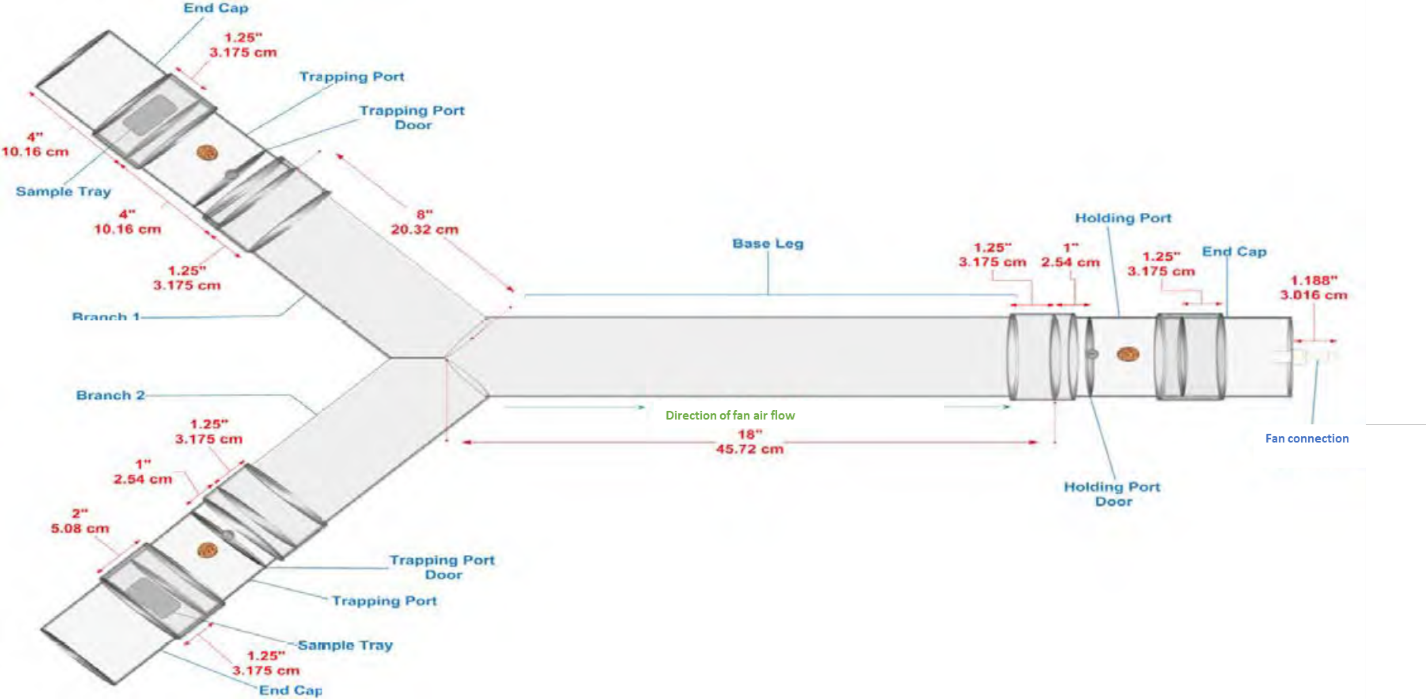


**Supplementary Figure S1**. Diagram and dimensions of the model Y-Tube olfactometer as given by WHO (2013) guidelines for efficacy testing of spatial repellents. Image reproduced and modified from WHO (2013).


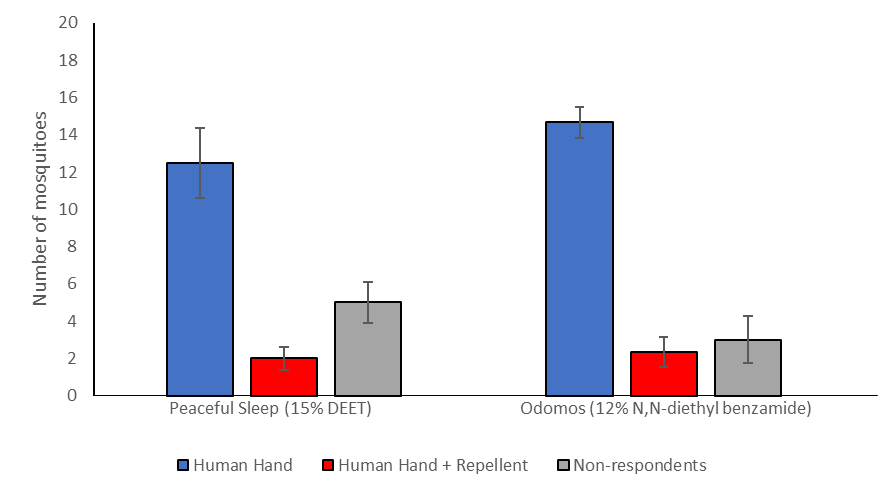


**Supplementary Figure S2.** Repellence efficacy of commercially available mosquito repellents using the Y-tube olfactometer. 18-20 mosquitoes were used for each replicate. A total of 6 replicates were used for each repellent. Numbers of non-respondent mosquitoes are included.


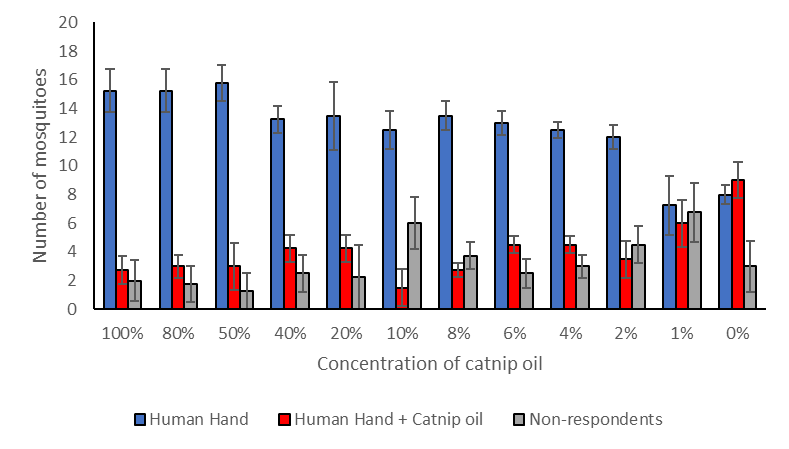


A


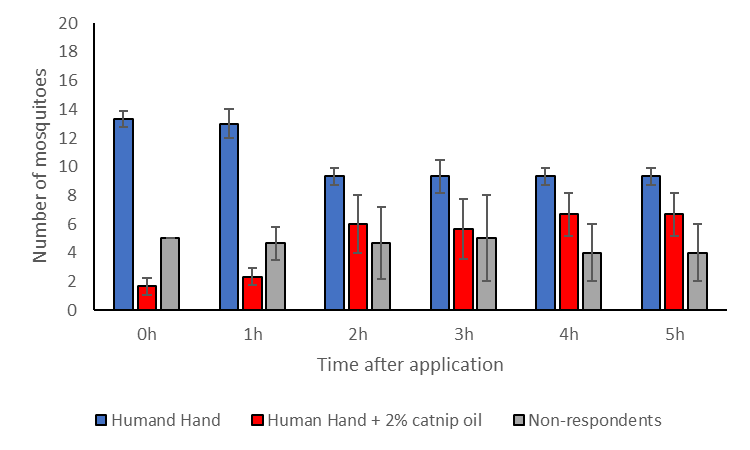


B

C


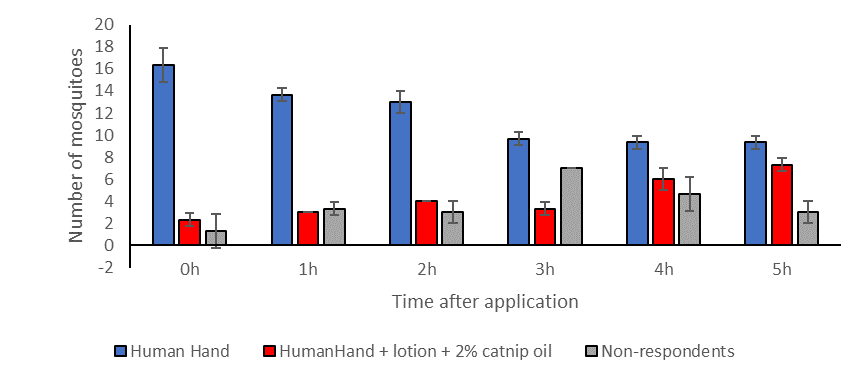


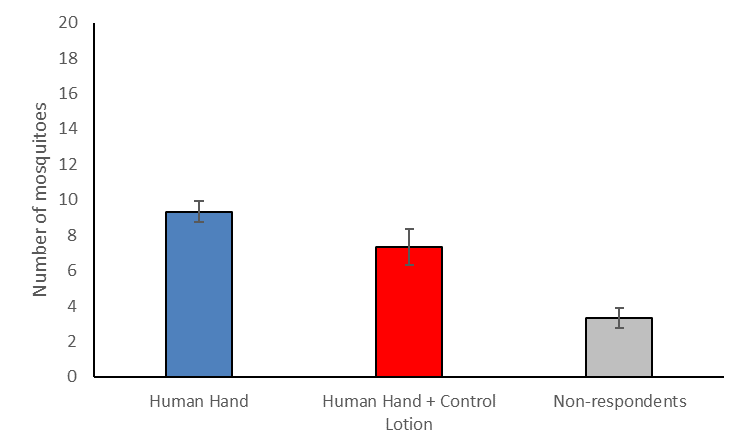


D

**Supplementary Figure S3.** Evaluation of catnip oil as a mosquito repellent, including non-responding mosquitoes. (A) Number of mosquitoes attracted to a human hand (no catnip oil, blue bars), human hand with catnip oil (red bars) applied at differing concentrations diluted to the appropriate concentration in olive oil, or remaining in the base leg/ holding port of the olfactometer (grey bars). 100% catnip oil had no olive oil diluent, 0% catnip oil was olive oil only. 20 mosquitoes were used for each replicate and 6 replicates were performed for each concentration. Numbers of non-responding mosquitoes are shown with grey bars. (B) Residual activity of 2% catnip oil diluted in olive oil over a 5-hour time-course post-application. 20 mosquitoes were used for each replicate and 3 replicates were performed for each concentration. (C) Residual activity of hand lotion supplemented with 2% catnip oil diluted in hand lotion over a 5-hour time-course post-application. 20 mosquitoes were used for each replicate and 3 replicates were performed for each concentration. Error bars represent standard deviations. (D) Number of mosquitoes attracted to the human hand vs human hand + control lotion lacking catnip oil, including non-responding mosquitoes. 20 mosquitoes were used for each replicate. A total of 3 replicates were performed. *t*-test was performed as per the Materials and Methods. No significant difference was observed.


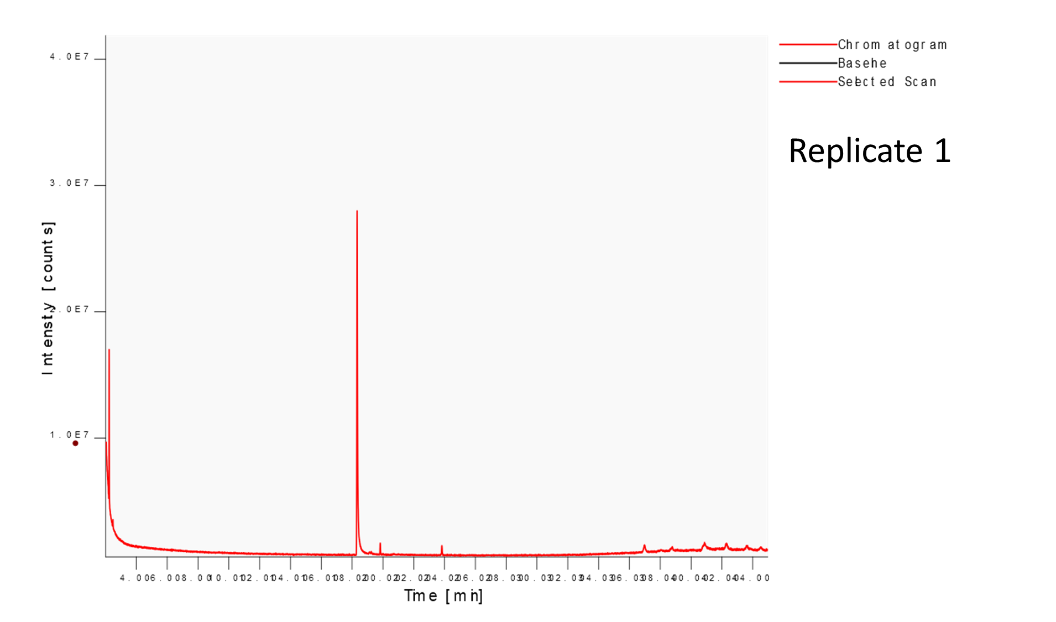


**
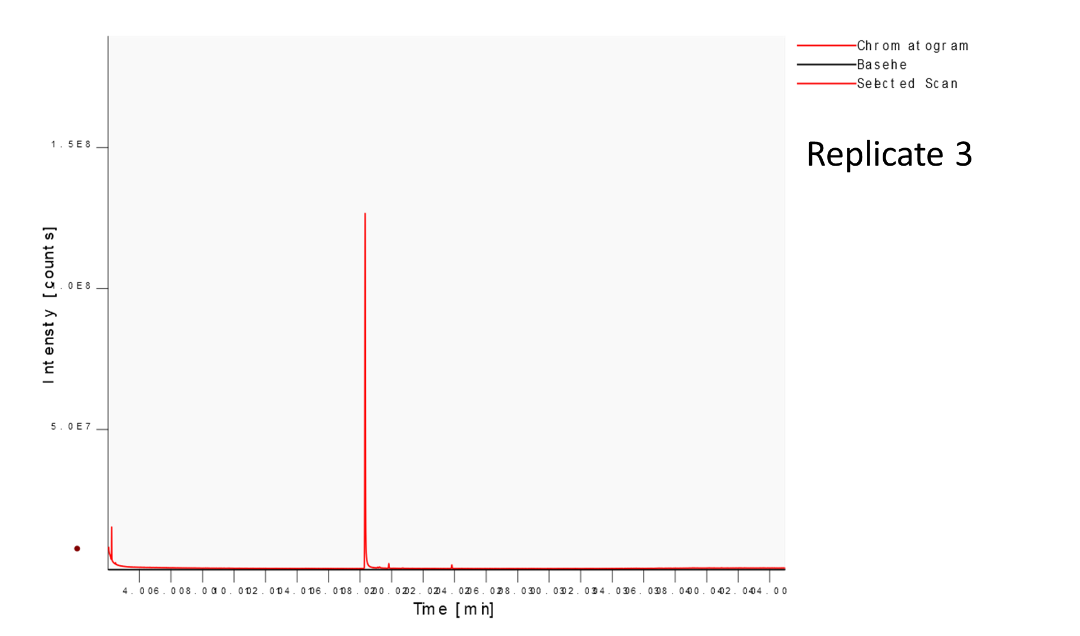

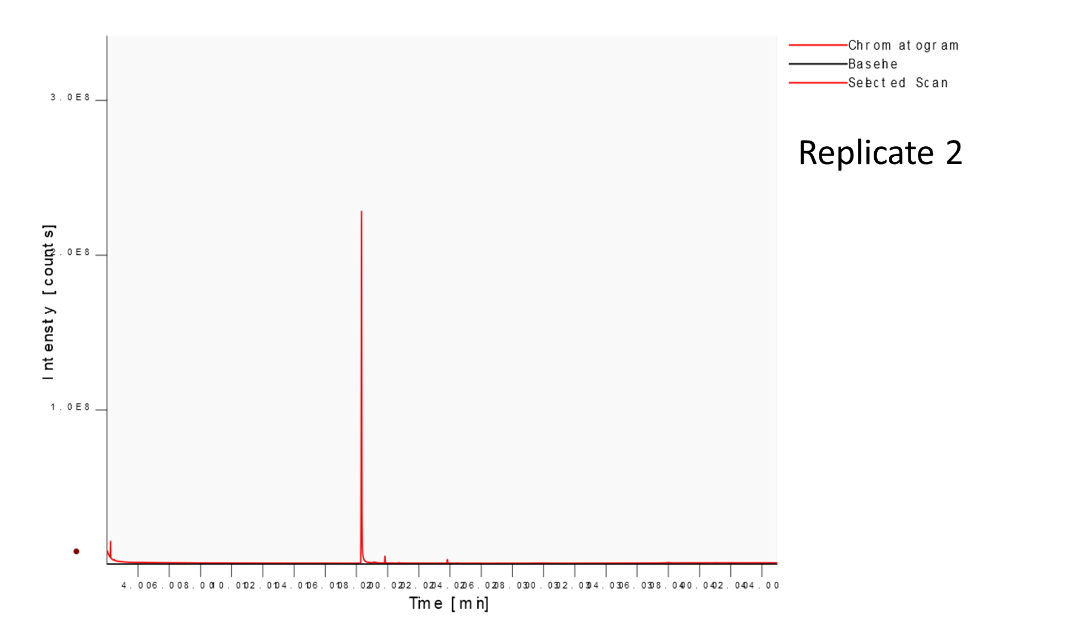
Supplementary Figure S4**. GC-MS chromatograms of each individual replicate of *Nepeta cataria* Chemotype A essential oil. Extraction and analysis is described in the Materials and Methods section.
